# Study on the clinical effect of percutaneous transforaminal endoscopic discectomy combined with annulus fibrosus repair in the treatment of single-segment lumbar disc herniation in young and middle-aged patients

**DOI:** 10.12669/pjms.40.3.3419

**Published:** 2024

**Authors:** Ya-fei Zhao, Bin-wu Tian, Qiu-shuang Ma, Meng Zhang

**Affiliations:** 1 Ya-fei Zhao, Department of Orthopedics, Baoding No.1 Hospital of Traditional Chinese Medicine, Baoding 071000, Hebei, P.R. China; 2 Bin-wu Tian, Department of Orthopedics, Baoding No.1 Hospital of Traditional Chinese Medicine, Baoding 071000, Hebei, P.R. China; 3 Qiu-shuang Ma, Department of Orthopedics, Baoding First Central Hospital, Baoding 071000, Hebei, P.R. China; 4 Meng Zhang, Department of Orthopedics, Baoding No.1 Hospital of Traditional Chinese Medicine, Baoding 071000, Hebei, P.R. China

**Keywords:** Percutaneous transforaminal endoscopic discectomy, Annulus fibrosus repair, Lumbar disc herniation

## Abstract

**Objective:**

To explore the clinical effect of percutaneous transforaminal endoscopic discectomy (PTED) combined with annulus fibrosus repair in the treatment of single-segment lumber disc herniation (LDH) in young and middle-aged patients.

**Methods:**

Ninty-six patients with single-segment LDH admitted to Baoding First Central Hospital from March 2021 to November 2022 were selected in the retrospective study. The patients were divided into endoscopic group and combined group according to different surgical methods. The surgical conditions, VAS score and ODI score the two groups of patients were compared, as well as the postoperative review results.

**Results:**

There were 50 patients in the endoscopic group the average operation time was 43.68 ± 10.77 minutes, the average intraoperative blood loss was 35.38 ± 10.02 ml, there were seven cases of surgical segment recurrence and 10 cases of postoperative intervertebral instability at the surgical segment. There were 46 patients in the combined group, the average operation time was 52.26 ± 8.39 minutes, the average intraoperative blood loss was 39.23 ± 9.02ml, there was one case of surgical segment recurrence and two cases of surgical segment intervertebral instability. The operation time (t=-4.328, P<0.01), postoperative recurrence cases (χ^2^=4.386, P<0.05) and intervertebral instability cases (χ^2^=5.366, P<0.05) of the two groups of patients). The difference was statistically significant. There was no significant difference in intraoperative blood loss between the two groups (t=-1.965, P>0.05). For six months after surgery, the differences in VAS and ODI scores between the two groups were statistically significant. In addition, there were statistically significant differences in the VAS scores and ODI scores of the two groups of patients at each time point after surgery compared with those before surgery (P<0.05).

**Conclusion:**

The clinical efficacy of PTED combined with annulus fibrosus repair showed better clinical efficacy than PTED alone, and it can reduce the occurrence of surgical segment recurrence and intervertebral instability, suggesting that PTED combined with annulus fibrosus repair may be worthy of promotion in clinical practice.

## INTRODUCTION

Lumbar disc herniation (LDH) is a common degenerative disease of the lumbar spine. Degeneration or damage of the annulus fibrosus and herniation or prolapse of the nucleus pulposus are the main causative factors of LDH, causing compression of the nerve roots, thecal sac or cauda equina, and the patient may A series of clinical symptoms such as low back pain and lower limb pain occur. For young and middle-aged LDH patients, conservative treatment is often used in clinical practice. When conservative treatment is ineffective and there are neurological symptoms^1^, surgical intervention is required. As age increases, the nucleus pulposus tissue begins to lose water and fibrous tissue gradually appears. However, the moisture content of the nucleus pulposus tissue in young and middle-aged people is higher, and they are more likely to have postoperative recurrence than elderly patients.^2^,^3^

Studies have shown that after discectomy, 0.5-7.9% of patients require re-operation to remove the intervertebral disc tissue that has degenerated and herniated again.^4^-^7^ In recent years, percutaneous transforaminal discectomy, as a minimally invasive surgery, has been widely used clinically and has become the main surgical method for the treatment of LDH. It has the advantages of small trauma, fast recovery, and the ability to preserve normal paravertebral and posterior vertebrae. Structure^8^. However, this surgical method also damages the annulus fibrosus and reduces the ability of the annulus fibrosus to repair itself, which leads to accelerated intervertebral disc degeneration and creates hidden dangers for the recurrence of LDH.^9^

In recent years, annulus fibrosus repair has received widespread attention from orthopedic surgeons. Studies have shown that repairing the annulus fibrosus during surgery can maintain the biological strength of the annulus fibrosus and reduce the recurrence of LDH. This study retrospectively analyzed the clinical data of young and middle-aged LDH patients treated in Baoding First Central Hospital, and compared the clinical efficacy and safety of percutaneous transforaminal endoscopic discectomy combined with surgical suture and PTED alone in the treatment of LDH.

## METHODS

From March 2021 to November 2022, 96 patients with single-segment LDH admitted to Baoding First Central Hospital were selected. The patients were divided into endoscopic group and combined group according to surgical methods, 50 patients treated with PTED alone were enrolled into the endoscopic group, and 46 patients treated with PTED combined with annulus fibrosus repair were enrolled into the combined group. Retrospective analysis of patient’s data, data were retrieved from the hospital information and management system to collect information of all patients.

### Ethical Approval:

The study was approved by the Institutional Ethics Committee of Baoding First Central Hospital, and written informed consent was obtained from all participants. (Date: July 28^th^ 2020.)

### Inclusion Criteria:

- Patients with age > 18 years old and < 50 years old;
- Patients with clearly diagnosed LDH with the presence of unilateral symptoms;
- Clinical signs consistent with imaging evidence;
- Patients with no obvious symptom improvement after conservative treatment for more than three months;
- Patients with follow-up time ≥ 1 year and complete follow-up data.

### Exclusion Criteria:

- Patients with age < 18 or > 50 years old;
- Patients with multiple-segment LDH;
- Patients complicated with other lumbar diseases, such as scoliosis;
- Patients with lumbar instability;
- Patients imaging evidence inconsistent with clinical signs;
- Patients with the presence of weakened muscle strength of lower limbs;
- Patients with a history of lumbar surgery.

Patient were managed with local anesthesia in prone position. Skin disinfection and sheet laying were routinely carried out. According to preoperative imaging data and clinical signs, transforaminal approach or interlaminar approach was selected. Generally, patients with protruding type, prolapsed type, and those with no distant nucleus pulposus will choose the translaminar approach, while those with lateral recess stenosis and severe spinal canal stenosis will use the transforaminal approach. Transforaminal approach: the surgical segment and the puncture distance were determined under fluoroscopy. The 18G puncture needle was punctured to the superior articular process to perform infiltration anesthesia on the facet joint. Puncture was carried out along the direction of the vertebral canal through the Kambin triangle, and the puncture was not stopped until the puncture needle point reached the anterioposterior position located at the midline of the spinous process and the lateral position located at posterior vertebral body line (PVBL) under the guidance of C-arm fluoroscopy.

The skin was cut by a sharp knife for about 0.7CM, a guide wire was inserted, soft tissues were expanded and opened step by step through a sleeve, and the foraminoplasty operation was carried out by using a trephine. The working channel was placed, adhesive tissues were separated under the endoscope, herniated intervertebral disc and nucleus pulposus tissues were excised, nerve roots were not fully decompressed until there was the visibility of pulsing of the nerve roots along with the pulse under the endoscope, and the skin was sutured after careful hemostasis and covered with sterile dressing.

### Interlaminar approach:

The surgical segment was determined under fluoroscopy. An incision (about 2cm) lateral to the spinous process was designed as a puncture point, the puncture needle was positioned at the inner edge of the interlaminar space under the confirmation of fluoroscopy, the skin was cut by the sharp knife for about 0.7cm, the expansion sleeve was placed along the guide needle, and finally the working channel was placed. Partial vertebral lamina of the inferior edge of superior lamina and the superior edge of inferior lamina inside the facet joint was subjected to abrasive drilling by a spherical abrasive drill under the endoscope. The ligamentum flavum was removed with a rongeur to expose the dura mater, the degenerated and protruded intervertebral disc and nucleus pulposus tissues were removed, followed by suturing the skin after careful hemostasis and covering the skin with sterile dressing.

Patients in the combined group were treated with the same procedures as the endoscopic group in terms of the transforaminal endoscopic operation. After completing the neurological decompression under the endoscope, the annulus fibrosus incision or tear was sutured using a disposable annulus fibrosus stapler. All surgical operations were performed by the same surgeon.

After the operation, the patient should stay in bed for 24 hours and wear a waistband to get out of bed and move appropriately. On the second day after the operation, rehabilitation training focusing on strengthening the core muscles of the lower back and lower limb muscles began, and three months after the operation Wear it inside the waistband and avoid heavy physical labor and strenuous exercise. Postoperative follow-up was conducted through outpatient review, telephone and WeChat. Patients were required to undergo outpatient or inpatient review one year after surgery, and all patients were followed up for more than one year.

### Observation Indexes:

- Record the patient’s surgical conditions, including operation time and intraoperative blood loss.
- Use the visual analogye scale (VAS) to evaluate the degree of pain in the lower limbs before surgery, one week after surgery, three months after surgery, six months after surgery, and one year after surgery. zero means no pain, ten means no pain. Divided into severe pain.
- The degree of neurological dysfunction was evaluated before operation, one week after operation, three months after operation, six months after operation and one year of follow-up with the use of Oswestry disability index (ODI).^10^
- One year after the operation, the patient came to the hospital for review, and lumbar spine X-ray and MRI were performed to record the recurrence of the surgical segment and the existence of intervertebral instability. Recurrence was defined as recurrence of herniated disc tissue and neurological symptoms at the operative level. Intervertebral instability was defined as vertebral body displacement >3 mm or angulation >15° on dynamic X-ray films.^8^

Statistical analysis was performed using SPSS 21.0 software. Measurement data consistent with normal distribution are expressed as mean ± standard deviation (χ ± s), and P value <0.05 is considered statistically significant. The measurement data between groups were tested for normality, and the measurement data consistent with normality were tested for independent samples t-test. Count data were compared using the χ^2^ test. Repeated measurement data within groups were subjected to analysis of variance.

## RESULTS

According to the inclusion and exclusion criteria, the clinical data of single-segment LDH patients admitted to Baoding First Central Hospital from March 2021 to November 2022 were retrospectively analyzed. A total of 96 patients were included in this study. The endoscopic group including 22 males and 28 females with an average age of 34.56±7.04 years old. There were 29 patients with left side symptoms, 21 patients with right side symptoms, two patients with L2-3 segment lesions, 14 patients with L3-4 segment lesions, 17 patients with L4-5 segment lesions and 17 patients with L5-S1 segment lesions. In addition, the combined group including 26 males and 20 females with an average age of 36.78±7.01 years old. There were 30 patients with left side symptoms, 16 patients with right side symptoms, one patient with L2-3 segment lesions, 11 patients with L3-4 segment lesions, 16 patients with L4-5 segment lesions and 18 patients with L5-S1 segment lesions. There was no significant difference in gender (χ^2^=1.503), age(*t*=-4.329), symptomatic side (χ^2^=0.527) and diseased segment (χ^2^=0.587) between the two groups (*P* > 0.05) (Table-I). The average operation time in the endoscopic group was 43.68±10.77 minutes, the average intraoperative blood loss was 35.38±10.02ml, there were seven cases of surgical segment recurrence, and 10 cases of surgical segment intervertebral instability. The average operation time in the combined group was 52.26±8.39 minutes, and the average intraoperative blood loss was 39.23±9.02ml. One patient had recurrence at the surgical segment, and two patients had intervertebral instability at the surgical segment.

**Table-I:** General information of patients in the two groups.

	Gender (Male/Female)	Age (Year)	Symptomatic Side (Left/Right)	Diseased Segments			

				L2-3	L3-4	L4-5	L5-S1
Endoscopic Group	22/28	34.56 ± 7.04	29/21	2	14	17	17
Combined Group	26/20	36.78 ± 7.01	30/16	1	11	16	18
Statistic Value	χ^2^=1.503	*T* =-4.329	χ^2^=0.527	χ^2^=0.587			
P Value	> 0.05	> 0.05	> 0.05	> 0.05			

There were significant differences in operation time (*t* =-4.328, *P*<0.01), recurrence cases (χ^2^= 4.386, *P*<0.05) and intervertebral instability cases (χ^2^=5.366, *P*<0.05) between the two groups, but there was no significant difference in intraoperative blood loss (*t*=-1.965, *P*>0.05) (Table-II). Six months after surgery, there was a statistically significant difference in the VAS and ODI scores between the two groups (P<0.05). The differences in VAS scores and ODI scores between the two groups at each time point were statistically significant (P<0.05), (Table-III).

**Table-II:** Comparisons in operation situation, postoperative reexamination results and intervertebral instability between the two groups.

	Operation time (min)	Intraoperative blood loss (ml)	Postoperative recurrence (n)	Intervertebral instability (n)
Endoscopic group	43.68 ± 10.77	35.38 ± 10.02	7	10
Combined group	52.26 ± 8.39	39.23 ± 9.02	1	2
Statistic value	*t* =-4.328	*t* =-1.975	χ^2^=4.386	χ^2^=5.366
*P* value	< 0.01	> 0.05	< 0.05	< 0.05

**Table-III:** Comparisons in VAS and ODI scores between the two groups

	Before operation	1 week after operation	3 months after operation	6 months after operation	1 year after operation	F	P
***VAS***							
Endoscopic group	7.68 ± 0.84	4.16 ± 0.98a	3.16 ± 0.92a	2.98 ± 0.82a	1.82 ± 0.66a	347.684	< 0.01
Combined group	7.67 ± 0.79	4.00 ± 0.76a	2.83 ± 0.74a	2.13± 0.54a	1.07 ± 0.25a	707.976	< 0.01
*t* value	-0.036	0.890	1.961	5.932	7.284		
*P* value	> 0.05	> 0.05	> 0.05	< 0.01	< 0.01		
***ODI***							
Endoscopic group	44.10 ± 1.30	54.20±1.68a	63.36± 2.22a	71.38±2.02a	74.34±2.11a	2166.998a	<0.01
Combined group	44.52 ± 1.17	54.87±1.72a	63.72± 1.96a	75.52±1.76a	87.46±1.97a	4313.287a	<0.01
Statistic value	-1.668	-1.929	-0.833	-10.671	-31.414		
*P* value	>0.05	> 0.05	> 0.05	< 0.01	< 0.01		

***Note:*** There was a significant difference when compared with preoperative VAS and ODI scores (*P* < 0.05).

## DISCUSSION

The results of this study showed that the postoperative recurrence rate of patients in the combined group was significantly lower than that in the endoscopic group (P<0.05). The reason is mainly that repairing and suturing the annulus fibrosus can close the crack, restore the mechanical integrity of the annulus fibrosus, maintain normal pressure in the intervertebral disc, reduce the re-herniation of the nucleus pulposus tissue, and at the same time maintain the intervertebral height to a certain extent, which is beneficial.

LDH is a common clinical disease, mostly seen in people aged 25-55 years. Most patients are effective with conservative treatment, but about 20% of patients still require surgical treatment. Open lumbar discectomy is the traditional surgical method for the treatment of LDH. However, Open surgery has higher requirements on the patient’s physical condition. The surgery will damage the spinal muscles and surrounding soft tissues, and there are also many postoperative complications. About 9%-32% of patients develop intractable low back pain after surgery.^11^ With the development of technology and the improvement of people’s requirements for quality of life, minimally invasive spine surgery has become a standard surgical procedure for the treatment of LDH.^12^ Among them, PTED is a minimally invasive surgical method commonly used in clinical practice. It has the advantages of short operation time, intraoperative With the advantages of less blood loss and quick postoperative recovery, it is increasingly used in the treatment of LDH in young and middle-aged people.^13^-^16^ However, some studies have shown that PTED only removes the intervertebral disc tissue without performing fusion surgery, which has the disadvantage of higher postoperative recurrence and repair rates than other surgical methods.^17^-^19^ The main reason is that the intervertebral disc tissue is destroyed by surgery. The biomechanical properties of the intervertebral disc may accelerate the degeneration of the intervertebral disc tissue, and the surgery will also cause damage to the annulus fibrosus. The unrepaired annulus fibrosus will also accelerate intervertebral disc degeneration, loss of intervertebral space height, and discogenic low back pain.

Studies have shown that repairing the annulus fibrosus can maintain the efficacy of surgery and reduce the risk of postoperative recurrence.^20^ The metabolism of intervertebral disc tissue avoids the occurrence of intervertebral instability and reduces the probability of postoperative recurrence.^21^,^22^ Moreover, the enhancement of intervertebral stability also avoids neurological symptoms and low back pain caused by intervertebral instability to a certain extent. The results of this study showed that at the end of the follow-up, the number of spinal instability cases in the endoscopic group was significantly higher than that in the combined group (P<0.05). This result is consistent with multiple clinical reports.^23^,^24^

The purpose of surgical treatment for LDH patients is to eliminate the patient’s clinical symptoms and improve the patient’s quality of life, and the VAS score and ODI score are currently commonly used observation items to evaluate the clinical effect of spine surgery.^25^,^26^ The results of this study showed that the postoperative VAS scores and ODI scores of the two groups of patients were significantly improved compared with those before surgery, and the VAS scores of the patients in the combined group were significantly lower than those of the endoscopic group six months after surgery (P<0.05), while the ODI scores were significantly lower than those before surgery. It was higher than that in the endoscopic group (P<0.05), indicating that endoscopic surgery combined with annulus fibrosus suturing can significantly improve the clinical treatment effect, and the patient’s prognosis is better than that of single disc removal, which is consistent with the results reported in multiple literatures.

### Limitations:

It includes small sample size and the follow-up time was also short and there is a lack of long-term follow-up data. Therefore, future studies with large sample sizes and long-term follow-up data are needed.

## CONCLUSION

We believe that PTED combined with annulus fibrosus repair can significantly improve surgical efficacy and patient prognosis, and its clinical results are better than PTED alone.

### Authors’ Contributions:

**YZ & BWT:** Designed this study and prepared the manuscript, are responsible and accountable for the accuracy and integrity of the work.

**QSM:** Collected and analyzed clinical data.

**MZ:** Significantly revised this manuscript.
